# Rapid ablation zone expansion amplifies north Greenland mass loss

**DOI:** 10.1126/sciadv.aaw0123

**Published:** 2019-09-04

**Authors:** Brice Noël, Willem Jan van de Berg, Stef Lhermitte, Michiel R. van den Broeke

**Affiliations:** 1 Institute for Marine and Atmospheric Research, Utrecht University, Utrecht, Netherlands.; 2 Department of Geoscience and Remote Sensing, Delft University of Technology, Delft, Netherlands.

## Abstract

Since the early 1990s, the Greenland ice sheet (GrIS) has been losing mass at an accelerating rate, primarily due to enhanced meltwater runoff following atmospheric warming. Here, we show that a pronounced latitudinal contrast exists in the GrIS response to recent warming. The ablation area in north Greenland expanded by 46%, almost twice as much as in the south (+25%), significantly increasing the relative contribution of the north to total GrIS mass loss. This latitudinal contrast originates from a different response to the recent change in large-scale Arctic summertime atmospheric circulation, promoting southwesterly advection of warm air toward the GrIS. In the southwest, persistent high atmospheric pressure reduced cloudiness, increasing runoff through enhanced absorption of solar radiation; in contrast, increased early-summer cloudiness in north Greenland enhanced atmospheric warming through decreased longwave heat loss. This triggered a rapid snowline retreat, causing early bare ice exposure, amplifying northern runoff.

## Teaser

Increased summer cloudiness triggers rapid ablation zone expansion in north Greenland, amplifying the mass loss.

## INTRODUCTION

Since the 1990s, mass loss from the Greenland ice sheet (GrIS) has significantly accelerated (*1*) due to increased glacial discharge (*2*) and decreased surface mass balance (SMB), the latter representing the difference between mass gains from snowfall and mass losses from meltwater runoff and sublimation. While glacial discharge strongly reacts to variations in ocean temperatures (*3*, *4*) and can regionally dominate mass loss in marine-terminating sectors of the ice sheet, i.e., particularly in northwest (NW) and southeast (SE) Greenland (*5*, *6*), the recent (1991–2015) GrIS-wide mass loss is primarily (~61%) ascribed to a decrease in SMB resulting from increased meltwater runoff (*7*), concurrent with the atmospheric warming that followed a recent summertime circulation change (*8*). Historically, the wide ablation zone in southwest (SW) Greenland contributed most (~32%) to the ice sheet runoff total (table S1), mainly driven by absorbed solar radiation at the dark bare ice surface in summer (*9*–*11*). However, especially over highly reflective snow surfaces, clouds can also enhance melt and runoff through reduced surface heat loss by longwave radiation (*12*, *13*). While the recent summertime circulation shift is responsible for reduced cloud cover over SW Greenland resulting in enhanced melt (*14*), to date, the physical processes responsible for the runoff changes in the north remain unclear.

**Fig. 1:**
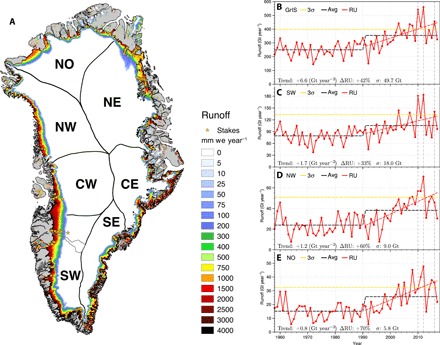
High-resolution runoff patterns and post-1990 changes. (**A**) Annual mean runoff (RU) over the GrIS and peripheral glaciers and ice caps (1958–2017) as modeled by RACMO2.3p2 and statistically downscaled to 1-km horizontal resolution. Yellow stars locate 213 stake sites where SMB is measured. The seven selected sectors of the GrIS derived from (*16*) are overlaid. (**B**) Time series of annual cumulative runoff integrated over the GrIS (red). Panels (**C**), (**D**), and (**E**) show the same for the SW, NW, and NO sectors, respectively, of the GrIS [see (A)]. Dashed red lines show linear runoff trends over 1991–2017, and dashed black lines show the averaged runoff over the periods 1958–1990 and 1991–2017, respectively. Dashed yellow lines mark exceptional runoff years, i.e., 3 SDs (σ) above the 1958–1990 mean. Dashed gray lines highlight recent exceptional runoff years over the GrIS, i.e., 2010, 2012, and 2016 (see fig. S7). Individual trends (1991–2017), relative increase in runoff post-1990 (%), and SD (σ) for the period 1958–1990 are listed at the bottom of each subpanel.

To address this, here, we combine SMB of a dedicated, high-resolution (5.5 km) run from the Regional Atmospheric Climate Model (RACMO2.3p2; fig. S1A), statistically downscaled to 1 km (Fig. 1A), with bare ice extent (BIE) derived from remotely sensed broadband shortwave clear sky albedo from the moderate resolution imaging spectroradiometer (MODIS) MCD43A3 500-m daily albedo product. This high resolution is essential to resolve the narrow ablation zone and marginal glacier tongues in sufficient detail (*15*), e.g., applying the statistical downscaling technique increases GrIS runoff by 25% compared to the original model resolution of 5.5 km (from 238 to 297 Gt year^−1^ for 1958–2017). Simulated GrIS climate and SMB components are evaluated using in situ SMB measurements collected in the accumulation (SMB > 0; 182 sites in fig. S1B) and ablation zones (SMB < 0; 213 sites in figs. S1C and S4A); meteorological observations recorded at 37 automatic weather stations (AWSs) from the Programme for Monitoring of the Greenland Ice Sheet (PROMICE), Institute for Marine and Atmospheric Research Utrecht (IMAU), and Greenland Climate Network (GC-Net) networks (figs. S2 and S3); and meltwater discharge measured at the Watson River in west Greenland (fig. S4B). The results show that RACMO2.3p2 realistically represents near-surface temperature, specific humidity, wind speed and air pressure (0.73 < *R*^2^ < 0.98), cloud conditions through shortwave and longwave radiation components (0.85 < *R*^2^ < 0.96), SMB (*R*^2^ = 0.78), and Watson River meltwater discharge (*R*^2^ = 0.91). We distinguish seven Greenland sectors (Fig. 1A)—NW, NO (north), NE (northeast), CE (center east), SE, SW, and CW (center west)—based on their distinct climatologies while covering similar areas (*16*). Not considering the very rugged SE and CE sectors, where bare ice area variability is well captured, although the absolute extent is underestimated, there is very good agreement between modeled and MODIS remotely sensed bare ice area (*R*^2^ = 0.82; fig. S5D). The model evaluation is discussed in more detail in Materials and Methods.

## RESULTS

### Increased runoff contribution from north Greenland

Figure 1A shows annual mean runoff for the period 1958–2017. Before 1990, the GrIS mass balance was near zero (*7*, *17*) or slightly negative (*16*), followed by accelerating mass loss. This mass loss is primarily driven by a 42% increase in runoff (1991–2017 versus 1958–1990), showing a positive trend of 6.6 ± 1.9 Gt year^−2^ (Fig. 1B), as compared to an estimated 4.4 ± 0.4 Gt year^−2^ trend in solid ice discharge (*16*). On the basis of a breakpoint analysis, we selected 1991 as the year after which the GrIS mass balance became predominantly negative (*7*). The frequency of extreme runoff years, i.e., at least 3 SDs (σ) above the 1958–1990 mean (dashed yellow lines in Fig. 1, B to E), increased from the 2000s onward when runoff reached a higher plateau (dashed black line). In GrIS sectors SW, NW, and NO, runoff during 1991–2017 increased by 33, 60, and 70%, respectively, relative to 1958–1990 (Fig. 1, C to E, and table S1). This shows that post-1990 runoff acceleration was relatively more pronounced in north Greenland than in south Greenland.

**Fig. 2:**
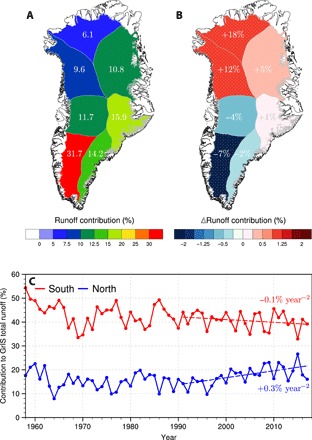
Regional changes in runoff contribution. (**A**) Average contribution of individual GrIS sectors to runoff totals for the period 1958–1990. (**B**) Post-1990 change in runoff contribution per sector (1991–2017 minus 1958–1990). Sectors experiencing significant change in runoff contribution, i.e., based on Student’s *t* test (*t* ≤ 0.10), are stippled with white dots. The post-1990 relative change in runoff contribution is also listed for each sector in white. (**C**) Time series of runoff contribution for North (blue; i.e., NO + NW) and South (red; i.e., CW + SW) Greenland. Post-1990 trends in runoff contribution are displayed as dashed lines.

Figure 2A shows that before 1991, the SW sector contributed 32% to the total runoff, mostly originating from the relatively wide marginal ablation zone exposing dark bare ice in summer (Fig. 1A), whereas the relatively cold and dry NW and NO sectors only contributed 10 and 6%, respectively (Fig. 2A and table S1). After 1991, runoff is significantly enhanced, but mostly so in NW (+60%) and NO (+70%) compared to +33% in SW (Fig. 1, B to E). As a result, the relative runoff contributions from NW and NO significantly increased by 12 and 18% (stippled in Fig. 2B; table S1). Because NW-NO and CW-SW Greenland show similar responses (Fig. 2B), these sectors are merged into two regions named “North” and “South.” Figure 2C highlights how the post-1990 trend in runoff contribution is negative, although insignificant (*P* = 0.193), in south Greenland (red) and significantly positive (*P* = 0.001) in north Greenland (blue; table S1).

### Rapid snowline retreat amplifies north Greenland runoff

**Fig. 3:**
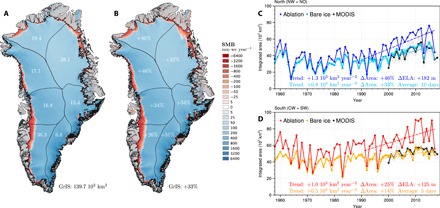
Rapid ablation zone expansion enhances runoff contribution from north Greenland. (**A**) Map of SMB averaged for the period 1958–1990. Numbers refer to the ablation zone area for individual sectors (10^3^ km^2^) and for the whole GrIS (bottom right). (**B**) Same as (A) for the period 1991–2017. Numbers refer to the relative ablation zone expansion (%) post-1990 for individual sectors and for the whole GrIS (bottom right). Time series of annual mean modeled ablation zone and summer bare ice area for (**C**) North Greenland (blue and cyan; i.e., NW + NO) and (**D**) South Greenland (red and orange; i.e., CW + SW sectors) over the period 1958–2017 compared to MODIS (black) bare ice area estimates (2000–2018). Dashed lines show the 1991–2017 trends. Numbers include trends, relative ablation/bare ice zone expansion, and change in ELA (i.e., SMB = 0) post-1990. In North and South Greenland, the modeled bare ice area is averaged over 10 and 5 days, respectively (see Materials and Methods). The cyan and yellow belt in (C) and (D) represents 1 SD of the 10 and 5 days used to estimate the modeled maximum bare ice area in North and South Greenland, respectively. The gray belt in (C) and (D) shows the uncertainty in measured MODIS bare ice area (see Eq. 1 in Materials and Methods).

We find that in concert with the runoff increase, the northern Greenland ablation zone expanded by 46% post-1990, almost twice as much as in the south (+25%; Fig. 3, A and B). At the same time, the bare ice zone, where the seasonal winter snow has completely melted at the end of summer, has grown more than twice as fast in the North (+33%) compared to the South (+14%), in good agreement with remotely sensed maximum BIE derived from MODIS (Fig. 3, C and D). Figure S6 shows how the SMB components changed in North and South Greenland for different ice sheet elevations. In the North, the snowline retreat moved the equilibrium line altitude (ELA; SMB = 0) upward by ~200 m from ~900 meters above sea level (masl) (fig. S6A) to ~1100 masl (fig. S6B), increasing runoff by 12 Gt year^−1^ (fig. S6C). In the South, the ELA migrated by ~100 m from ~1350 masl (fig. S6D) to ~1450 masl (fig. S6E), increasing runoff by ~17 Gt year^−1^ (red line in fig. S6F). We conclude that the faster snowline retreat in the North is responsible for its enhanced contribution to Greenland runoff totals (Fig. 2B). Note also how a saturated firn percolation zone with net ablation (SMB < 0; difference between the ablation zone and bare ice area in Fig. 3C) progressively forms in the North after the mid-1990s, resembling more and more the southern GrIS ablation zone (Fig. 3D).

### Circulation change increases early-summer cloudiness in north Greenland

**Fig. 4:**
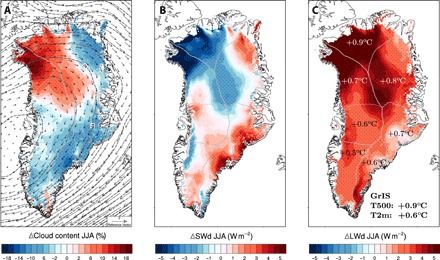
Recent shift in summer atmospheric circulation and impact on cloudiness. (**A**) Post-1990 change in summer cloud content (JJA; 1991–2017 minus 1958–1990) as modeled by RACMO2.3p2 at 5.5 km. Changes in large-scale circulation (black vectors; see inset for wind speed estimation) and in height of the 500-hPa geopotential (dashed black lines) are overlaid. (**B**) Change in modeled SWd and (**C**) LWd radiation. In (C), sector-averaged near-surface temperature (2 m) increase is displayed in black and white for southern and northern Greenland, respectively. Average GrIS-wide temperature increase at 500 hPa and at 2-m altitude is listed in the inset. The seven GrIS sectors are outlined in gray in (A) to (C). Stipples highlight regions showing a significant change, i.e., > 1 SD of the 1958-1990 period (σ) in (A) cloud content (σ = 4 g m^−2^), (B) SWd (σ = 1.9 W m^−2^), and (C) LWd (σ = 1.7 W m^−2^).

We relate the latitudinal contrast in runoff increase to the recent change in the large-scale summertime atmospheric circulation. The latter change, observed in the Arctic from the late 1990s onward (*8*), promoted southwesterly advection of warm air from Baffin Bay toward the GrIS. Figure 4A illustrates the post-1990 change in June-July-August (JJA; 1991–2017 minus 1958–1990) large-scale circulation over Greenland (contours and arrows). A persistent high-pressure ridge results in an anticyclonic circulation anomaly over the GrIS (vectors in Fig. 4A). As a result, the NO and NW sectors experience anomalous westerly advection of warm and humid air from Baffin Bay, increasing summer (JJA) cloud content after 1991 by, respectively, 5 and 10% on average (3 and 8 ± 4 g m^−2^) and up to 20% locally (29 ± 4 g m^−2^). In contrast, northerly advection of relatively dry inland air reduces cloud content over the southern GrIS by 2 to 6% on average (−3 to −6 ± 4 g m^−2^), in line with (*14*). Clouds play a pivotal role in the GrIS melt climate by modulating downward shortwave (SWd) and longwave (LWd) radiation fluxes (*18*). Figure 4B shows post-1990 changes in summer SWd (JJA; 1991–2017 minus 1958–1990), mirroring the large-scale changes in clouds: SWd decreases in the NW (−2.3 ± 1.9 W m^−2^ on average) and NO (−3.2 ± 1.9 W m^−2^) sectors, while it mostly increases elsewhere (Fig. 4B). In contrast, LWd has increased everywhere by 3.5 W m^−2^ on average (Fig. 4C) due to overall higher free atmosphere temperatures (+0.9°C at 500 hPa; see inset in Fig. 4C). However, the LWd increase peaks in NW (4.7 ± 1.7 W m^−2^) and in NO (5.3 ± 1.7 W m^−2^) sectors, where increased clouds further enhance LWd compared to, e.g., SW Greenland (2.6 ± 1.7 W m^−2^). Therefore, the near-surface temperature increase is amplified in northern Greenland (e.g., +0.9 ± 0.1°C for NO) relative to other sectors (e.g., +0.5 ± 0.1°C for SW). Note the marked longitudinal contrast in cloud content change between NO-NW and NE sectors, where cloud content is reduced by 2% on average and down to 14% locally (−1 g m^−2^ down to −10 ± 4 g m^−2^). This is due to a large-scale Foehn effect, resulting in relatively warm and dry air flowing down the lee side of the main northern ice divide, after having generated enhanced orographic precipitation at the upwind slopes of the NW and NO sectors. The average circulation and radiative flux anomalies shown in Fig. 4 represent large interannual variability (fig. S7) that reflects the exact position of the high-pressure ridge in summer (*19*). As a result, runoff also shows increased interannual variability after 1991 (Fig. 1, B to E).

### Increased early-summer cloudiness triggers rapid northern snowline retreat

**Fig. 5:**
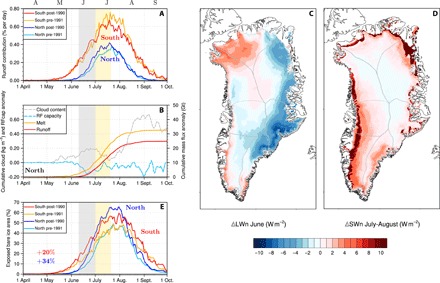
Increased early-summer cloudiness triggers runoff amplification in north Greenland. (**A**) Time series of daily (April-September) mean runoff contribution to GrIS totals for periods 1958–1990 and 1991–2017 in South (orange and red; i.e., CW + SW sectors) and North (cyan and blue; i.e., NO + NW sectors) regions. (**B**) Time series of daily (April-September) cumulative anomalies (1991–2017 minus 1958–1990) in surface melt (orange) runoff (red) for the North region (NO + NW sectors; right *y* axis). Dashed gray and cyan lines (left *y* axis) show cumulative anomalies in cloud content and refreezing capacity, i.e., the fraction of meltwater and rainwater retained and/or refrozen in the firn. Anomalies in (**C**) June LWn and (**D**) July-August SWn. (**E**) Daily exposed bare ice area post-1990 and pre-1991 for the South (red and orange) and North (blue and cyan) regions expressed as a fraction (%) of the ablation zone area. Numbers at the bottom left corner of (E) express the relative increase (%) in maximum bare ice area post-1990 for North (blue) and South (red) Greenland. In (A), (B), and (E), the gray and yellow shades outline the period during which runoff contribution of North Greenland significantly increases (A) under high and low cloudiness successively (B).

Figure 5 shows how anomalies in climate and runoff are interacting during the course of the melt season (April-September). Figure 5A shows the daily runoff contribution of North and South regions to GrIS total (% per day for the period April-September) averaged for 1958–1990 (cyan and orange lines) and 1991–2017 (blue and red lines). Figure 5B displays the cumulative anomalies (1991–2017 minus 1958–1990) in melt (orange; Gt) and runoff (red) as well as in cloud content (dashed gray; kg m^−2^) and refreezing capacity (dashed cyan; unitless), i.e., the fraction of meltwater and rainwater that is retained and/or refrozen within the firn pack. Figure S8 shows similar results for the South region. Figure 5 (C and D) shows the anomalies in June net longwave (LWn) and July-August net shortwave radiation (SWn). Figure 5E shows the daily fraction of the ablation zone area exposing bare ice in summer for North and South Greenland for the period before (cyan and orange) and after 1991 (blue and red). Note that, in a warming climate in which the equilibrium line migrates upward, the ablation zone comprises both the marginal bare ice area and the lower percolation zone, where saturated firn contributes to meltwater runoff in late summer.

In the South, the decrease in runoff contribution, i.e., the difference between the red and orange solid lines in Fig. 5A, occurs mainly during the peak melt season between July and early August (yellow shade in fig. S8). In that period, the cumulative cloud content anomaly gradually decreases and becomes negative (i.e., less clouds compared to pre-1991) from mid-May and throughout the melt season (fig. S8), enhancing SWd (Fig. 4B). The post-1990 melt and runoff increase, estimated at 46 and 37 Gt, is driven by increasing SWn (Fig. 5D): Earlier removal of the seasonal snow cover exposes dark bare ice over the extensive South ablation zone, enhancing the absorption of incoming solar radiation (*10*). In contrast, increased runoff contribution in North Greenland after 1991, i.e., the difference between the blue and cyan solid lines in Fig. 5A, starts early in the melt season in June and ends in late July. This increase is concurrent with high cloud content in June (gray shade in Fig. 5B) followed by low cloud content until late July (yellow shade in Fig. 5B). In early summer, increased cloudiness and atmospheric temperatures in North Greenland (Fig. 4, A and C) act in concert to warm the snow-covered surface through reduced longwave heat loss (Fig. 5C). This warming triggers an early melting of the shallow snow cover, rapidly migrating the snowline further inland. As a result, early exposure of dark bare ice causes a rapid decline in surface albedo (fig. S9A) and reduces meltwater retention (fig. S9B). At higher elevations, the presence of summer clouds maintains firn temperatures close to the melting point during nighttime (*13*), preventing refreezing of percolating meltwater; note the sharp decline in firn refreezing capacity that mirrors the cloud content increase (gray shade; Fig. 5B and fig. S9B). Figure 5E highlights the rapid snowline retreat in the North, resulting in an expansion of the (maximum) daily bare ice area by 34% compared to 20% in the South. Early-summer exposure of bare ice further leads to anomalous high absorption of incoming shortwave radiation for reduced cloud content in July (yellow shade; Fig. 5, B and D). This chain of events significantly amplifies the relative runoff increase in North Greenland (+63%; table S1) compared to South Greenland (+34%) and other sectors (fig. S9C).

## DISCUSSION

The whole of Greenland has warmed since the early 1990s, but mostly in the north, amplifying northern mass loss through enhanced meltwater runoff. Using a combination of remote sensing and output of a regional climate model, we show that this latitudinal gradient can be coupled to a circulation shift that brings more clouds to northern Greenland. This triggered early snowline retreat in the dry north, causing a rapid expansion of the bare ice (+33%) and ablation zones (+46%), twice as fast as in the south (+14 and +25%). As a result, North Greenland has significantly increased its relative contribution to total GrIS mass loss through enhanced runoff. Superimposed on increased melt, rising temperatures and increased cloudiness in the North also led to a summer rainfall increase of ~42%, contributing 5 Gt year^−1^ or 8% to North Greenland runoff totals (fig. S6, A to C). In late summer (September), rainfall in North Greenland occasionally even equals runoff over bare ice, in line with (*20*). If the current trends in ablation area expansion were to continue, the northern ablation zone would equal the size of the southern counterpart in another ~45 years. This would require the current circulation anomaly to persist, but predicting this is highly uncertain, as Earth System Models (ESMs) from the fifth phase of the Coupled Model Intercomparison Project (CMIP5) fail to reproduce the contemporary large-scale Arctic circulation change (*21*) that led to the latitudinal gradient in runoff response reported here. This highlights the importance of better resolving Arctic circulation variability and cloud microphysics in ESMs (*12*, *13*), e.g., cloud phase, water content, and optical thickness, to obtain reliable GrIS mass change projections.

## MATERIALS AND METHODS

### Regional atmospheric climate model

We used a new run at 5.5-km horizontal resolution of the polar (p) version of RACMO2.3p2 for the period 1958–2017. For detailed description of the model and recent updates, we refer to (*22*). In brief, RACMO2.3p2 incorporates the dynamical core of the High Resolution Limited Area Model (*23*) and the physics from the European Centre for Medium-Range Weather Forecasts–Integrated Forecast System (ECMWF-IFS cycle CY33r1) (*24*). RACMO2.3p2 includes a multilayer snow module that simulates melt, water percolation, and retention in snow, refreezing, and runoff (*25*). The model also accounts for dry snow densification (*26*), and drifting snow erosion and sublimation (*27*). Snow albedo is calculated on the basis of snow grain size, cloud optical thickness, solar zenith angle, and impurity concentration in snow (*28*). Compared to (*22*), no model physics have been changed. However, increased horizontal resolution of the host model, i.e., 5.5 km instead of 11 km, better resolves gradients in SMB components over the topographically complex ice sheet margins and neighboring peripheral glaciers and ice caps.

### Initialization and setup

Figure S1A shows the model integration domain. RACMO2.3p2 was forced at its lateral boundaries by ERA-40 (1958–1978) (*29*) and ERA-Interim (1979–2017) (*30*) re-analyses on a 6-hourly basis within a 24–grid cell wide relaxation zone (fig. S1A). The forcing consists of temperature, specific humidity, pressure, wind speed, and direction being prescribed at each of the 40 vertical atmosphere model levels. Upper atmosphere relaxation (nudging) was also implemented in RACMO2.3p2 (*31*). The model has 40 active snow layers that were initialized in September 1957 using temperature and density profiles derived from the offline IMAU Firn Densification Model (IMAU–FDM) (*26*). Glacier outlines and surface topography were prescribed from a down-sampled version of the 90-m Greenland Ice Mapping Project (GIMP) Digital Elevation Model (DEM) (*32*). Bare ice albedo was prescribed from the 500-m MODIS 16-day albedo product (MCD43A3), as the 5% lowest surface albedo records for the period 2000–2015, minimized at 0.30 for dark bare ice in the low-lying ablation zone, and maximized at 0.55 for bright ice under perennial snow cover in the accumulation zone.

### Climate evaluation

We evaluated the modeled GrIS present-day climate by comparing modeled meteorological variables (fig. S2), i.e., 2-m air temperature and specific humidity, 10-m wind speed and surface pressure, and radiative fluxes (fig. S3), i.e., short/longwave down/upward radiation (SWd, LWd, SWu, and LWu, respectively), to daily measurements collected at 37 AWS (green dots in fig. S1A). These AWSs are operated by the PROMICE (18 sites for 2007–2016) (*33*), by the IMAU (5 sites for 2004–2016) (*34*), and by the GC-Net (14 sites for 1995–2017) (*35*). Erroneous radiation measurements, caused by, e.g., sensor riming in winter and sensor heating in summer, were discarded from the analysis following the method described in (*22*). Sites showing an elevation difference of >100 m compared to the GIMP DEM down-sampled to 5.5-km resolution were not used (nine sites). Because of frequent data gaps and sensor issues, the GC-Net results for air temperature, specific humidity, wind speed, and surface pressure are included in fig. S2 for completeness but were not used for evaluation statistics.

Figures S2 and S3 show that RACMO2.3p2 at 5.5 km agrees well when compared to daily measurements of meteorological variables and radiative fluxes. Notably, 2-m temperature and specific humidity only show small biases of 0.14°C and −0.11 g kg^−1^, respectively. Modeled and observed radiative fluxes also agree well with small biases, e.g., 4.3 and −7.8 W m^−2^ for SWd and LWd, respectively. Note that these biases can be partly ascribed to sensor uncertainty, estimated at 2.7 and 1.2% for daily mean SWd (4.8 W m^−2^) and LWd (2.7 W m^−2^) radiation (*34*). Additional uncertainty originates from window heating in summer, i.e., shortwave-induced sensor heating, potentially overestimating LWd records by 10 to 15 W m^−2^ on a 6-min basis (*34*) or less than 5 W m^−2^ on daily average. Similar evaluations in four individual GrIS sectors (*22*) demonstrated that modeled SWd and LWd were as well represented in northern Greenland as in other regions. The strong correlations with remotely sensed (*13*) and in situ SWd (fig. S3A) and LWd (fig. S3C) demonstrate that RACMO2.3p2 realistically represents Greenland cloud characteristics. Furthermore, errors in modeled SWd/LWd [root mean square error (RMSE) of ~20 W m^−2^] were much smaller than the difference between clear sky and overcast conditions, reaching ~100 W m^−2^ (*12*). Note that surface pressure shows systematic biases at several stations, resulting from an up to 100-m difference between the model and station elevation.

### SMB evaluation and statistical downscaling

For SMB evaluation, we used 182 accumulation measurements from stakes, firn pits, and cores (*36*) derived from airborne radar campaign (*37*) in the GrIS accumulation zone (white dots in fig. S1A) as well as 1073 ablation measurements from 213 stake sites (yellow dots in fig. S1A) (*38*). Figure S1B compares modeled (5.5 km in blue and 11 km in red) and observed SMB in the accumulation zone. Compared to the previous simulation at 11-km resolution, the 5.5-km run slightly improves the SMB representation in the accumulation zone, with a smaller bias and RMSE reduced by 4.5 and 2.7 mm we (millimeters water equivalent) or 21 and 4%, respectively. Relative to (*22*), significant improvements were found in the ablation zone, where RACMO2.3p2 at 5.5 km now explains 48% of the observed SMB variance (*R*^2^) instead of 41% previously, with a smaller bias and RMSE reduced by 230 and 140 mm we or 38 and 11%, respectively. This is due to the better resolved SMB patterns over narrow glaciers and marginal ablation zones at a resolution of 5.5 km (fig. S1A) compared to 11 km [figure 1 of (*22*)].

Although improving on previous model versions, significant SMB biases persist locally in the low ablation zone, where surface ablation exceeds 4 m we year^−1^ (fig. S1C). For instance, at stake QAS_L (291 masl), i.e., the lowest site of the 30-km-long Qagssimiut transect at the southern tip of the GrIS, strong winter precipitation and summer ablation gradients are not well resolved, resulting in SMB biases of up to 8 m we for summer 2010 (fig. S1C). In these rugged marginal regions, a spatial resolution of 5.5 km remains insufficient to resolve narrow glaciers and confined ablation zones that are prime contributors to GrIS runoff totals (*15*). To address this, we applied statistical downscaling to the 5.5-km product as described in (*15*, *39*), which corrects runoff for biases in elevation and bare ice albedo. This allows us to reproduce more accurately the high runoff rates observed at the GrIS margins, significantly improving the agreement with SMB measurements (fig. S4A). The downscaled product explains 78% of the SMB variance (*R*^2^), with a positive bias of 70 mm we, suggesting a small runoff underestimation. In addition, a comparison between modeled and observed meltwater discharge from the Watson River catchment in SW Greenland (gray contour in sector SW of Fig. 1A) also shows good agreement despite a small negative bias of, on average, ~0.4 Gt year^−1^ (or km^3^) (1976–2016) and up to ~2 Gt year^−1^ for exceptional melt episodes that occurred in summers of 2010 and 2012 (fig. S4B). The average runoff bias reaches 5% for the period 1976–2016, peaking at ~20% for extreme runoff years (2010 and 2012), in line with (*40*). On the basis of this, we used a conservative 20% runoff uncertainty for annual basin integrated values. Good agreement between modeled and in situ surface ablation (fig. S4A), i.e., primarily driven by runoff, as well as with measured meltwater discharge from the Watson River in west Greenland (fig. S4B), confirms that meltwater runoff is well reproduced by RACMO2.3p2 (0.78 < *R*^2^ < 0.91). Last, a recent comparison between the period 2003–2017 mass changes derived from the Gravity Recovery and Climate Experiment (GRACE) and from a combination of modeled RACMO2.3p2 SMB at 1-km resolution and estimated solid ice discharge showed good agreement on both GrIS-wide and individual basin scales (*6*).

### MODIS bare ice area

Remotely sensed annual bare ice area was derived from broadband shortwave clear sky albedo data from the MCD43A3 MODIS 500-m daily albedo product (http://dx.doi.org/10.5067/MODIS/MCD43A3.006). To discard invalid albedo records, we masked erratic albedo grid cells from the GrIS-wide, daily 16-day MCD43A3 MODIS product (2000–2018), resorting to full Bidirectional Reflectance Distribution Function (BRDF) inversions. Valid daily MODIS data were classified as ice- or snow-covered grid cells using an upper threshold for shortwave albedo of 0.60 (SW_0.60_), slightly higher than the assumed maximum albedo of bright bare ice (0.55). The justification for this is to ensure that the maximum bare ice area is fully captured by MODIS. Subsequently, the daily ice/snow cells were converted to annual bare ice area assuming that the bare ice is exposed in summer if (i) the current pixel is classified as ice at least 5 days in that year (5th percentile) and (ii) that pixel is located within the long-term modeled ablation zone of the GrIS (SMB < 0; 1991–2017), i.e., including both the bare ice and lower percolation zone below the long-term ELA of ~1450 masl. These criteria allow the elimination of spurious bare ice conditions, e.g., meltwater lakes, meltwater runoff streams, and superimposed ice, that are detected at higher elevations in the percolation zone of the GrIS. For instance, in summer 2012, MODIS detected bare ice conditions at AWS S9 (1520 masl) located close to the equilibrium line along the K-transect in western Greenland (67°N), whereas fieldwork reported superimposed ice conditions resulting from firn saturation. Masked pixels were then filled on the basis of the recurrence of ice/snow observations over 2000–2018 for that cell. In brief, masked pixels exposing ice more than 50% of the time for the period 2000–2018 are assumed bare ice. This method may result in bare ice area overestimation in early years of MODIS operation, which includes more invalid albedo estimates. Alternatively, daily bare ice conditions could have been extrapolated onto masked pixels based on surrounding grid cells. However, we discarded this approach because (i) local MODIS pixels may not be representative of surrounding areas and (ii) a large number of MODIS records were masked due to, e.g., a large solar zenith angle and local cloud cover, deteriorating the quality of local MODIS estimates. Extrapolation may thus strongly overestimate remotely sensed (observed) bare ice area for the entire 2000–2018 period. That is why we used the ice recurrence method to obtain a spatially continuous annual BIE product covering the whole of the GrIS for the period 2000–2018 (fig. S5A). The MODIS product is sensitive to the shortwave albedo threshold used to discriminate between ice and snow conditions. To reflect this, we estimated an uncertainty in maximum MODIS BIE by repeating the analysis described above using the assumed maximum albedo of bright bare ice of 0.55 (SW_0.55_) as an upper threshold. The BIE uncertainty was estimated GrIS wide and for individual sectors as follows

$$\mathrm{Uncertainty}\;=\;{{\mathrm{BIE}}_{\mathrm{SW}}}_{0.60}-{{\mathrm{BIE}}_{\mathrm{SW}}}_{0.55}$$ (1)

The latter uncertainty was used to derive a symmetric error band around the MODIS bare ice area estimates in Fig. 3 (C and D) and fig. S5 (B to D).

### RACMO2.3p2 bare ice area evaluation

For model evaluation that is consistent with the MODIS product, we estimated a 10-day bare ice area in northern sectors (NW, NO, and NE) and a 5-day one for southern regions. Daily modeled bare ice area was estimated as the integrated area of pixels showing a surface albedo of ≤0.55 on the original 5.5-km grid, bilinearly interpolated to the 1-km GIMP ice mask. The 10 and 5 largest daily estimates of bare ice area are averaged for North and South Greenland, respectively, to obtain a maximum annual bare ice area comparable to MODIS data. This is deemed reasonable as MODIS records 16-day albedo and because less valid MODIS albedo estimates are available for the North (55% of valid records, on average, for the period 2000–2018) compared to the South (79%). This difference stems from large solar zenith angles at high latitudes, negatively affecting the quality of the satellite measurements. Figure S5A shows minimum and maximum annual BIE derived from MODIS for the period 2000–2018, i.e., summer of 2000 (black) and 2012 (red). Modeled GrIS bare ice area (orange) agrees well with MODIS (black; fig. S5B), although with a negative bias of ~8100 km^2^ (fig. S5C) peaking for exceptional melt years, e.g., 2012, 2014, and 2016. This negative bias, i.e., up to 16,100 km^2^ in 2014, mainly originates from the CE (~25%) and SE (~25%) sectors of the GrIS (fig. S5D). One possible explanation is that, in CE and SE sectors, RACMO2.3p2 simulates too much snowfall in winter, delaying or preventing the exposure of bare ice in summer. At the same time, these two sectors also experience frequent overcast conditions, negatively affecting the quality of the MODIS bare ice product. In other sectors, RACMO2.3p2 agrees well with observed bare ice area (*R*^2^ = 0.82; fig. S5D) with a small negative bias (140 km^2^).

## Supplementary Material

http://advances.sciencemag.org/cgi/content/full/5/9/eaaw0123/DC1

Download PDF

Rapid ablation zone expansion amplifies north Greenland mas los

## SUPPLEMENTARY MATERIALS

Supplementary material for this article is available at http://advances.sciencemag.org/cgi/content/full/5/9/eaaw0123/DC1 Table S1. Changes in runoff production and contribution per sector. Fig. S1. RACMO2.3p2 integration domain and SMB evaluation. Fig. S2. Evaluation of modeled meteorological variables at 5.5 km. Fig. S3. Evaluation of radiative fluxes at 5.5 km. Fig. S4. Evaluation of the downscaled product using in situ and catchment measurements. Fig. S5. Evaluation of the modeled bare ice area using remote sensing. Fig. S6. Post-1990 upward migration of the equilibrium line. Fig. S7. High interannual variability in summer atmospheric circulation and impacts on the cloudiness. Fig. S8. Reduced summer cloud cover enhances melt in southern Greenland. Fig. S9. Post-1990 changes in surface conditions.
