# Supplementary material for: Rapid ablation zone expansion amplifies north Greenland mass loss
Source: Sci Adv. 2019 Sep 4;5(9):eaaw0123. doi: 10.1126/sciadv.aaw0123 (PMC6726448; doi:10.1126/sciadv.aaw0123)
Supplement: http://advances.sciencemag.org/cgi/content/full/5/9/eaaw0123/DC1 [file supp_5_9_eaaw0123__index.html]

Science Advances | Science AdvancesAAASSearchScience AdvancesMenu

## Supplementary Materials

**This PDF file includes:**

- Table S1. Changes in runoff production and contribution per sector.
- Fig. S1. RACMO2.3p2 integration domain and SMB evaluation.
- Fig. S2. Evaluation of modeled meteorological variables at 5.5 km.
- Fig. S3. Evaluation of radiative fluxes at 5.5 km.
- Fig. S4. Evaluation of the downscaled product using in situ and catchment measurements.
- Fig. S5. Evaluation of the modeled bare ice area using remote sensing.
- Fig. S6. Post-1990 upward migration of the equilibrium line.
- Fig. S7. High interannual variability in summer atmospheric circulation and impacts on the cloudiness.
- Fig. S8. Reduced summer cloud cover enhances melt in southern Greenland.
- Fig. S9. Post-1990 changes in surface conditions.

Download PDF

**Files in this Data Supplement:**

- Adobe PDF - aaw0123\_SM.pdf
