# Supplementary material for: Rapid ablation zone expansion amplifies north Greenland mass loss
Source: Sci Adv. 2019 Sep 4;5(9):eaaw0123. doi: 10.1126/sciadv.aaw0123 (PMC6726448; doi:10.1126/sciadv.aaw0123)
Supplement: Download PDF [file aaw0123_SM.pdf]

Science Advances
AAAS

advances.sciencemag.org/cgi/content/full/5/9/eaaw0123/DC1

Supplementary Materials for

Rapid ablation zone expansion amplifies north Greenland mass loss

Brice Noël*, Willem Jan van de Berg, Stef Lhermitte, Michiel R. van den Broeke

*Corresponding author. Email: b.p.y.noel@uu.nl

Published 4 September 2019, *Sci. Adv.* **5**, eaaw0123 (2019)
DOI: 10.1126/sciadv.aaw0123

This PDF file includes:

Table S1. Changes in runoff production and contribution per sector.
Fig. S1. RACMO2.3p2 integration domain and SMB evaluation.
Fig. S2. Evaluation of modeled meteorological variables at 5.5 km.
Fig. S3. Evaluation of radiative fluxes at 5.5 km.
Fig. S4. Evaluation of the downscaled product using in situ and catchment measurements.
Fig. S5. Evaluation of the modeled bare ice area using remote sensing.
Fig. S6. Post-1990 upward migration of the equilibrium line.
Fig. S7. High interannual variability in summer atmospheric circulation and impacts on the cloudiness.
Fig. S8. Reduced summer cloud cover enhances melt in southern Greenland.
Fig. S9. Post-1990 changes in surface conditions.

Table S1. Changes in runoff production and contribution per sector. Annual mean runoff production (Top) and contribution (Bottom) for the periods 1958-1990 and 1991-2017, as well as relative changes (1991-2017 minus 1958-1990; %) and recent trends (1991-2017) for individual sectors, the whole GrIS and the North (NO+NW) and South (CW+SW) regions. The uncertainty in runoff production is estimated at 20% and the uncertainty in runoff contribution is estimated as one standard deviation of the period 1958-1990 (resp. 1991-2017).

Runoff	Units	NW	NO	NE	CW	CE	SW	SE	GrIS	North	South
Mean 58-90	Gt yr^{-1}	23.9±4.8	15.2±3.0	27.1±5.4	29.3±5.9	39.7±7.9	79.2±15.8	35.5±7.1	250.0±50.0	39.1±7.8	108.5±21.7
Mean 91-17	Gt yr^{-1}	38.2±7.6	25.6±5.1	40.3±8.1	39.6±7.9	56.5±11.3	105.5±21.1	48.5±9.7	354.5±70.9	63.8±12.8	145.1±29.0
Δ	%	+59.8	+69.6	+48.8	+35.4	+42.3	+33.2	+36.5	+41.8	+63.2	+33.7
Trend 91-17	Gt yr^{-2}	+1.2	+0.8	+1.0	+0.7	+0.8	+1.7	+0.4	+6.6	+2.0	2.4
**Contribution**	**Units**	**NW**	**NO**	**NE**	**CW**	**CE**	**SW**	**SE**	**GrIS**	**North**	**South**
Mean 58-90	% yr^{-1}	9.6 ±2.2	6.1 ±1.7	10.8±2.2	11.7±1.7	15.9±2.9	31.7±3.9	14.2±2.3	-	15.7±3.9	43.3±3.5
Mean 91-17	% yr^{-1}	10.7±2.4	7.2±1.8	11.3±2.1	11.2±1.1	16.1±2.1	29.6±3.0	13.9±2.2	-	17.9±4.2	40.8±4.1
Δ	%	+11.5	+18.0	+4.6	-4.3	+1.3	-6.6	-2.1	-	+12.4	-6.0
Trend 91-17	% yr^{-2}	+0.2	+0.1	+0.1	0.0	-0.1	-0.1	-0.2	-	+0.3	-0.1

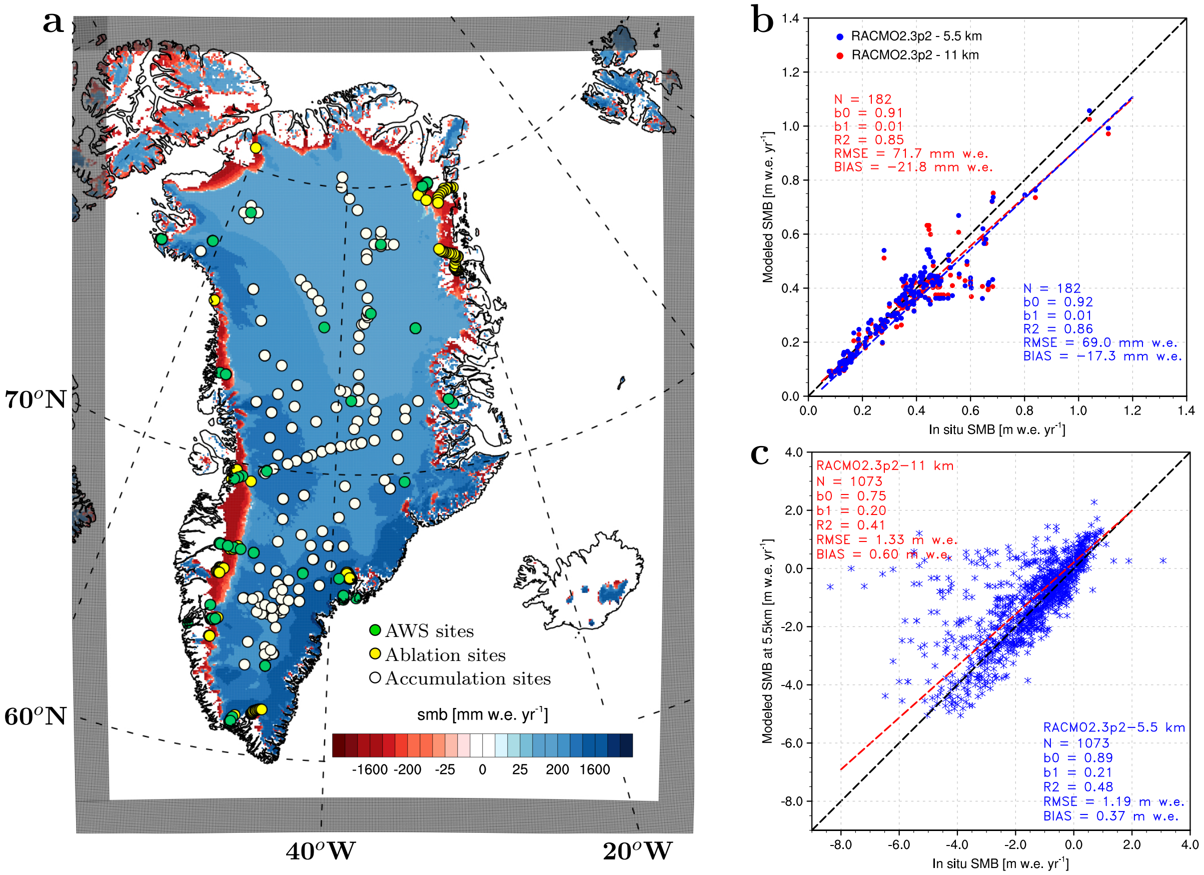

Fig. S1. RACMO2.3p2 integration domain and SMB evaluation. (a) Annual mean SMB as modeled by RACMO2.3p2 at 5.5 km horizontal resolution averaged for the period 1958-2017. Black dots delineate the relaxation zone (24 grid cells) where the model is forced by ERA re-analyses. Green dots locate 37 AWS used for model evaluation: based on meteorological variables (fig. S2) and radiative fluxes (fig. S3). Comparison of modeled and observed SMB at (b) 182 accumulation sites (white dots in fig. S1a) and (c) 213 ablation sites (yellow dots in fig. S1a). Figure S1b compares accumulation from the previous 11 km (red dots) with the new 5.5 km (blue dots) versions of RACMO2.3p2; regression lines (dashed) and statistics, i.e. number of observations (N), regression slope (b0), intercept (b1), determination coefficient (R^2), RMSE and bias, are displayed for the 11 km (red) and 5.5 km (blue) simulations. Figure S1c also displays statistics from the previous 11 km (red) and new 5.5 km (blue) model simulations over the ablation zone; the regression line (red dashed line) is based on the 5.5 km product (blue stars).

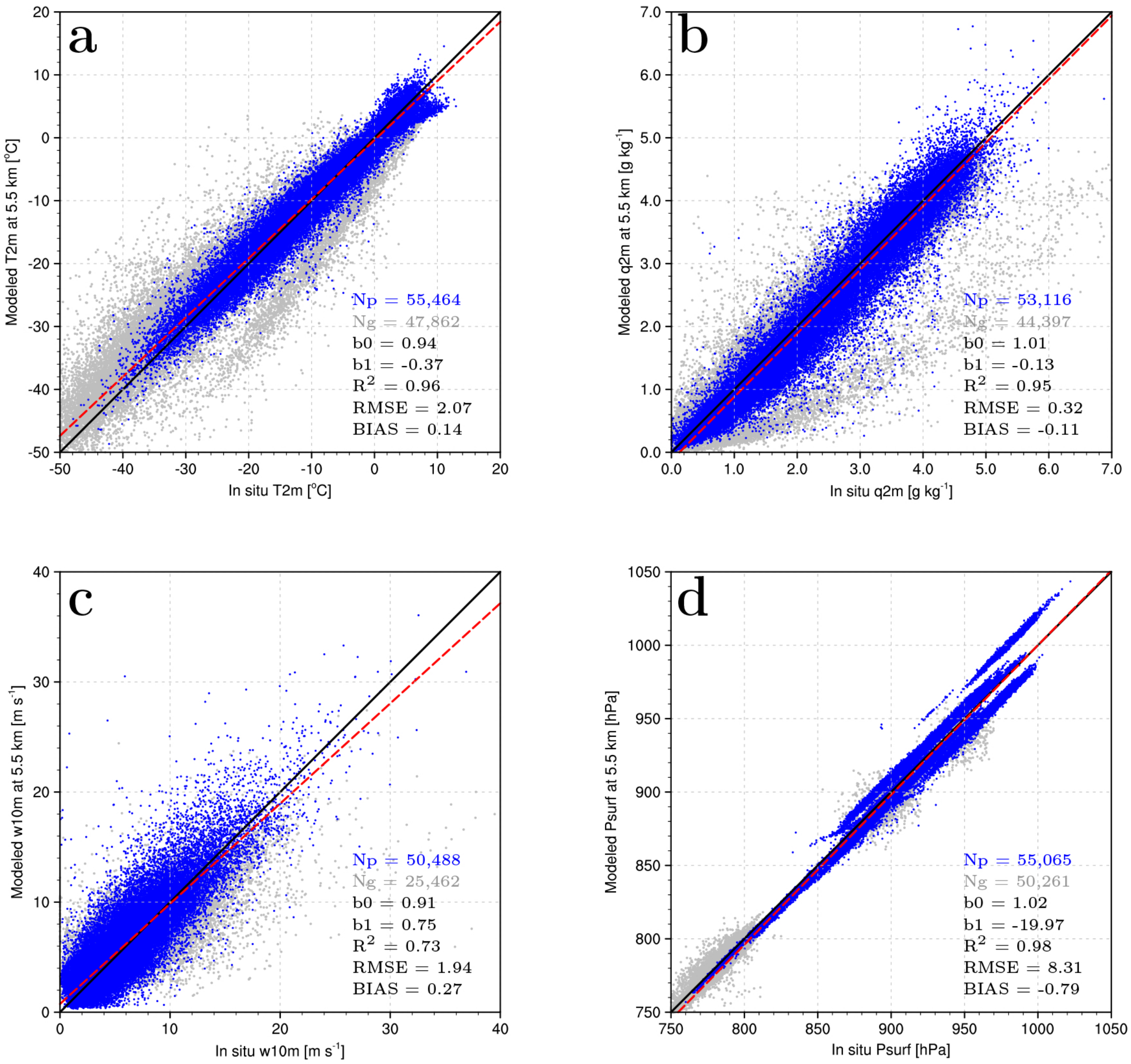

Fig. S2. Evaluation of modeled meteorological variables at 5.5 km. Comparison between modeled (5.5 km) and observed (a) 2 m temperature (t_{2m}, ^{o}C), (b) 2 m specific humidity (q_{2m}, g kg^{-1}), (c) 10 m wind speed (w_{10m}, m s^{-1}) and (d) surface pressure (Psurf, hPa) collected at 37 AWS (green dots in fig. S1a). For each variable, the linear regression including all records is displayed as red dashed line. Statistics including number of PROMICE-IMAU records (Np; blue dots) and number of GC-Net records (Ng; grey dots), the linear regression slope (b0) and intercept (b1), determination coefficient (R^2), RMSE and bias are listed for each variable. Statistics are based on comparison with PROMICE-IMAU data only.

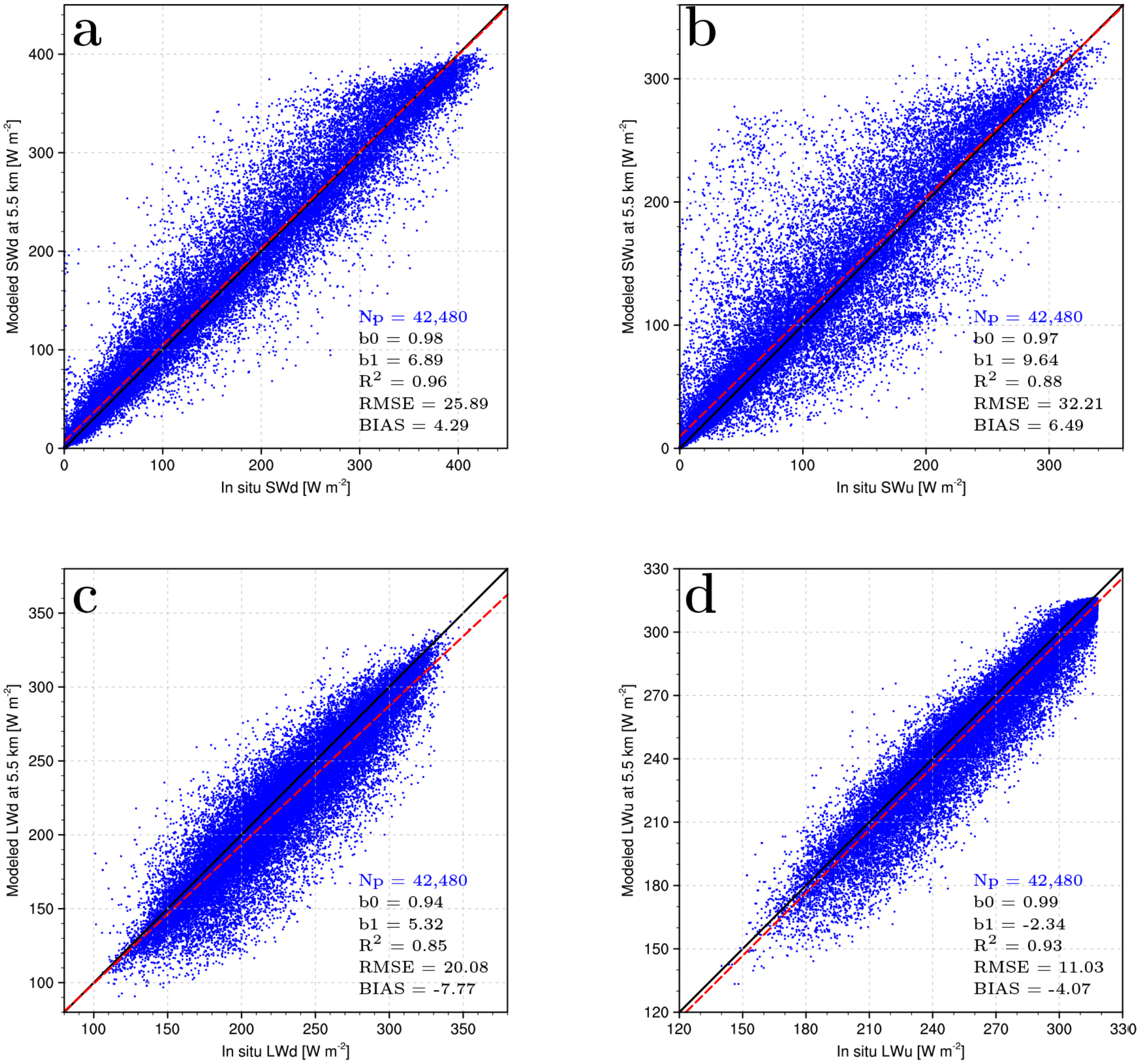

Fig. S3. Evaluation of radiative fluxes at 5.5 km. Comparison between daily average modeled (5.5 km) and observed (a) shortwave downward, (b) shortwave upward, (c) longwave downward and (d) longwave upward radiation (W m^{-2}) collected at 23 AWS (green dots in fig. S1a). For each variable, regression including all records is displayed as red dashed line. Statistics including number of PROMICE-IMAU records (Np; blue dots), regression slope (b0) and intercept (b1), determination coefficient (R^2), RMSE and bias are listed for each variable.

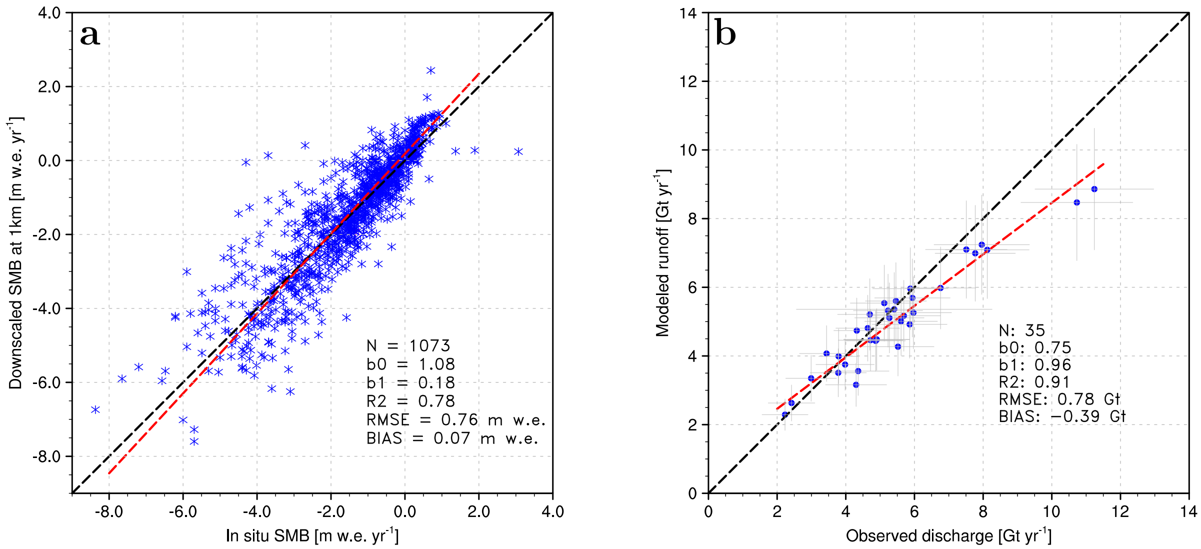

Fig. S4. Evaluation of the downscaled product using in situ and catchment measurements. (a) Comparison between annual downscaled (1 km) and measured SMB at ablation stake sites located at the GrIS margins (1958-2015). (b) Comparison between measured and downscaled annual meltwater discharge from the Watson River catchment (grey contour in Fig. 1a; 1976-2016). Uncertainty bars for observed and modeled (20%) discharge are displayed in grey. In (a-c) statistics including number of observations (N), determination coefficient (R^2), slope (b0) and intercept (b1) of the regression line (red dashed). Root Mean Square Error (RMSE) and bias are also listed.

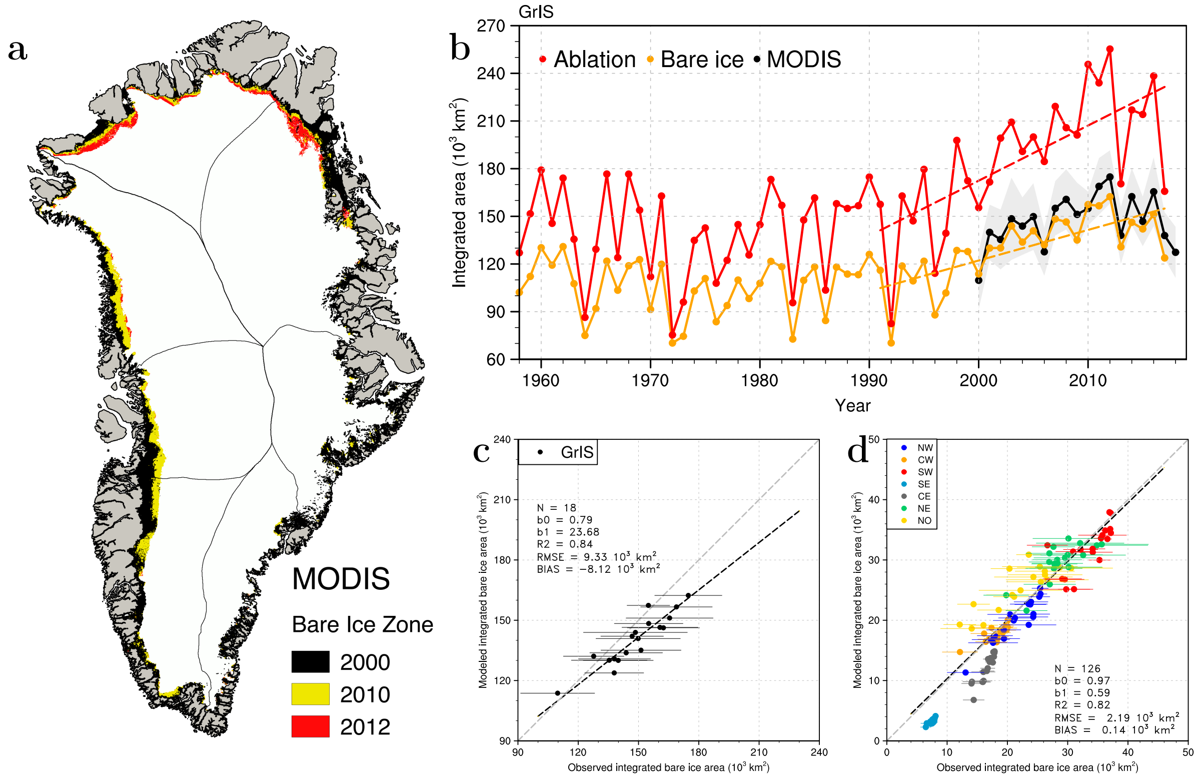

Fig. S5. Evaluation of the modeled bare ice area using remote sensing. (a) Maximum bare ice extent derived from MODIS records in summer 2000 (black), 2010 (yellow) and 2012 (red). (b) Time series of the GrIS ablation zone area (red), modeled (orange) and observed annual bare ice area (MODIS; black) for the period 1958-2017. The grey belt in fig. S5b shows the uncertainty in MODIS bare ice area (see Eq. 1 in the Material and Methods Section). Scatter plot comparing modeled and observed bare ice area for the period 2000-2017 (c) over the whole of the GrIS and (d) for individual sectors. Error bars represent the estimated uncertainty in MODIS bare ice area. In (c-d) statistics including number of observations (N), determination coefficient (R^2), slope (b0) and intercept (b1) of the regression line (red dashed), Root Mean Square Error (RMSE) and bias. In (d) statistics and regression line are estimated excluding CE and SE sectors.

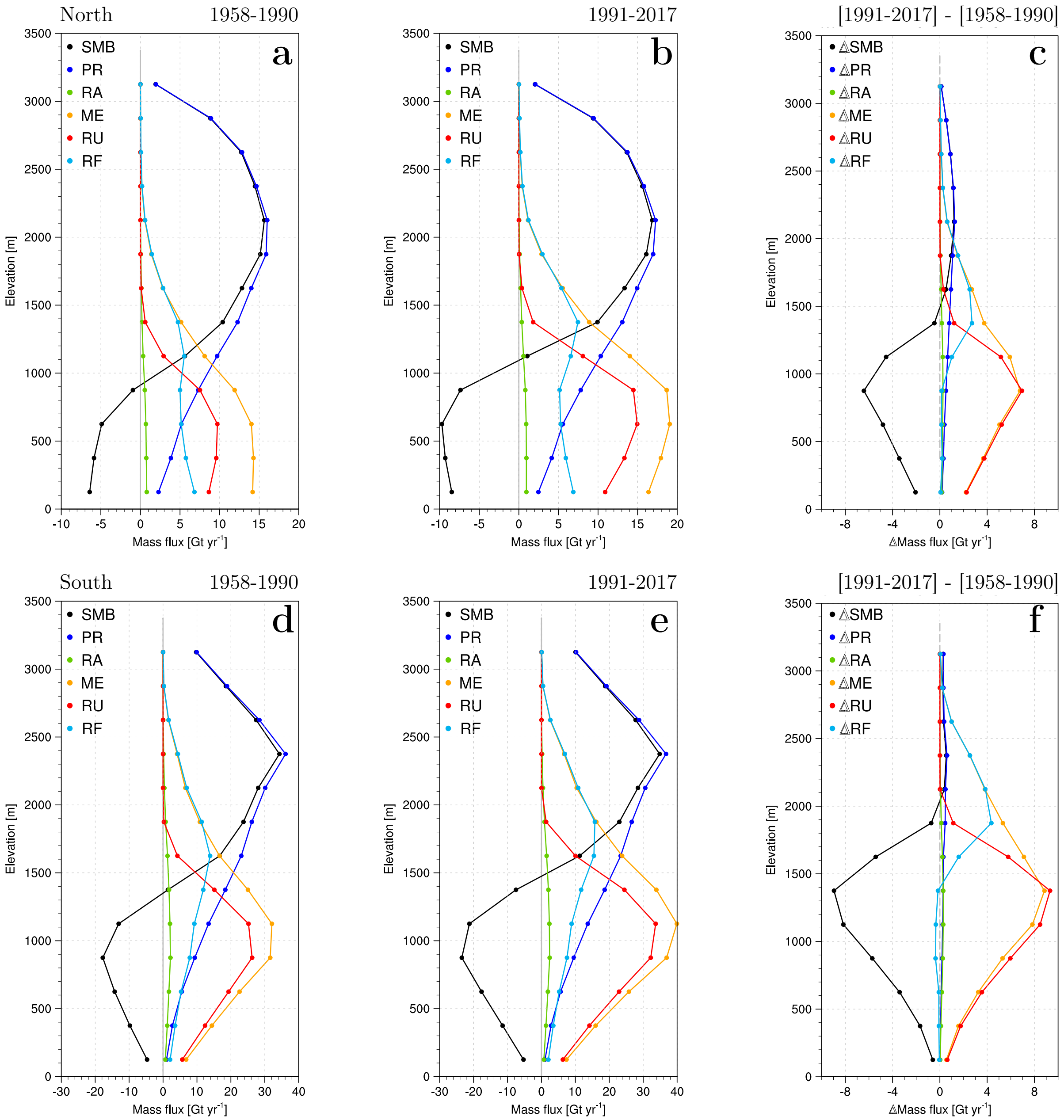

Fig. S6. Post-1990 upward migration of the equilibrium line. Vertical profiles of surface mass fluxes integrated per elevation bin (250 m) over North (upper row, i.e. NW + NO sectors) and South (lower row, i.e. CW + SW sectors) regions for the period 1958-1990 (a and d), 1991-2017 (b and e) and the difference between the two periods (1991-2017 minus 1958-1990, c and f). Mass fluxes include SMB (black), total precipitation (rain and snow; blue), rainfall (green), melt (orange), runoff (red), refreezing and retention (cyan).

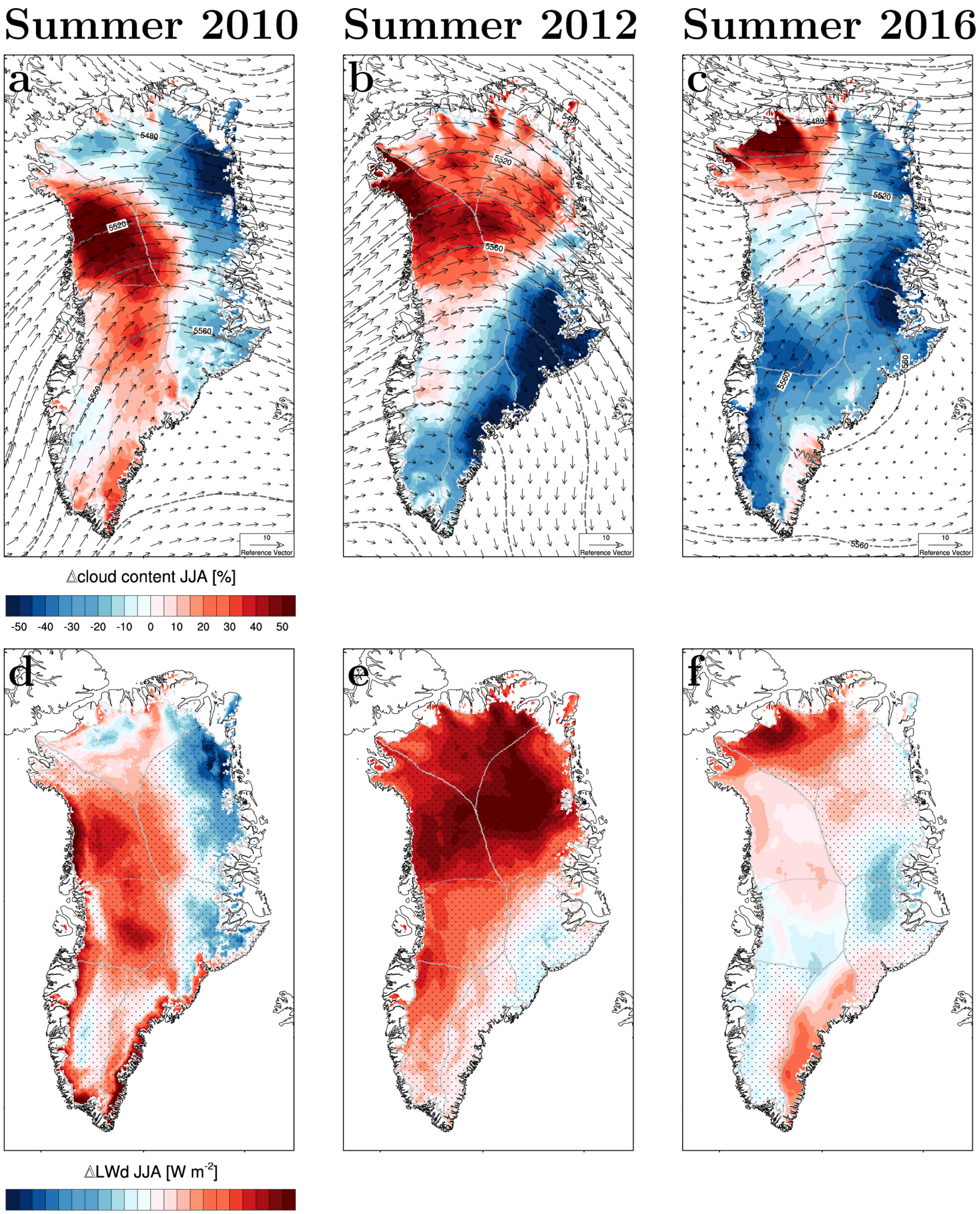

Summer 2010
Summer 2012
Summer 2016
a
b
c
5480
5520
5560
10
Reference Vector
Δcloud content JJA [%]
-50 -40 -30 -20 -10 0 10 20 30 40 50
d
e
f
ΔLWd JJA [W m-2]
-20 -16 -12 -8 -4 0 4 8 12 16 20

Fig. S7. High interannual variability in summer atmospheric circulation and impacts on the cloudiness. (a) Anomalies in cloud content for summer 2010 (JJA; 2010 minus 1958-1990). Overlaid are the JJA mean atmospheric circulation in 2010 (black vectors, see inset for estimating wind speed) and height of the 500 hPa geopotential (dashed black lines). (d) Anomalies in longwave downward radiation for summer 2010. GrIS sectors experiencing exceptional runoff in 2010, i.e. three times as large as the 1958-1990 standard deviation, are stippled with black dots. (b-e) and (c-f) same as (a-d) but for summers of 2012 and 2016, respectively. Note that the location of the high-pressure ridge not only affects the summer circulation but also local cloudiness (figs. S7a-c). For instance, reduced cloud content in NO Greenland during summer 2010 resulted in high but not exceptional meltwater runoff, i.e. below three standard deviations (figs. 1e and S7d), while other sectors did experience extreme runoff in that summer. The same holds for NW Greenland in summer 2016 (figs. 1d and S7f). Conversely, increased cloud content in the NO sector in summers of 2012 and 2016 triggered exceptional runoff episodes, i.e. above three standard deviations (Fig. 1e), concurrent with large positive LWd anomalies (figs. S7e,f).

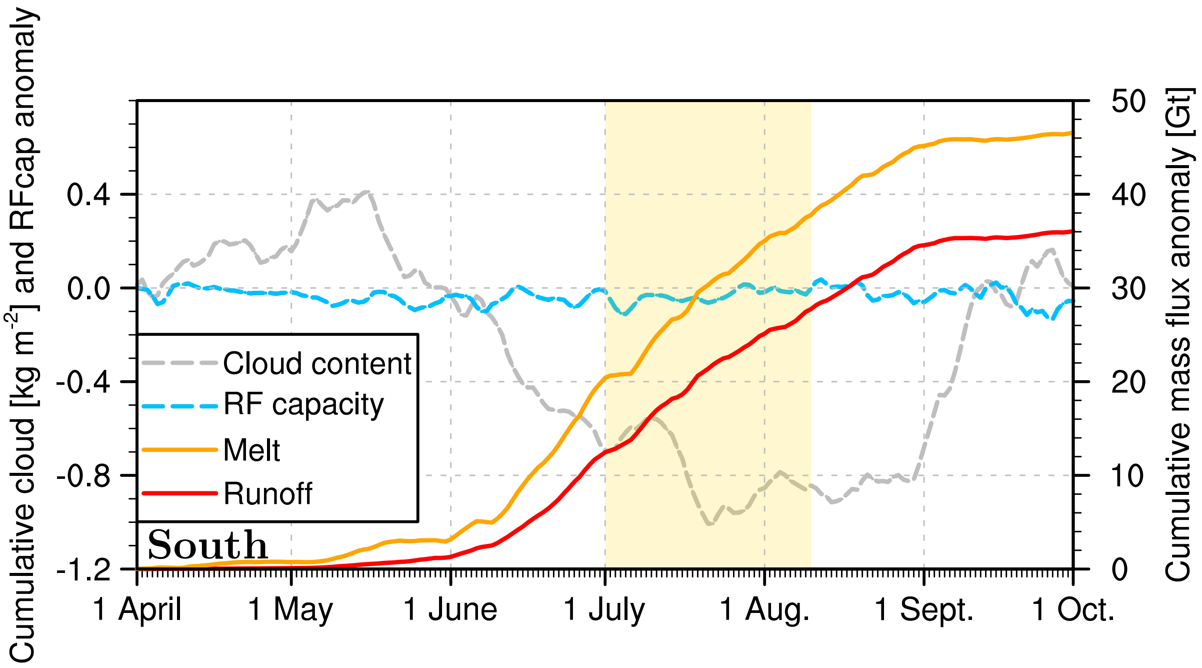

Fig. S8. Reduced summer cloud cover enhances melt in southern Greenland. Time series of daily (April-September) cumulative anomalies (1991-2017 minus 1958-1990) in surface melt (orange), runoff (red) for the South region (CW + SW sectors; right y-axis). Dashed grey and cyan lines (left y-axis) show cumulative anomalies in cloud content and refreezing capacity, i.e. the fraction of melt and rain water retained and/or refrozen in the firn. The yellow shade outlines the period during which runoff contribution of South Greenland significantly declines for reduced cloudiness.

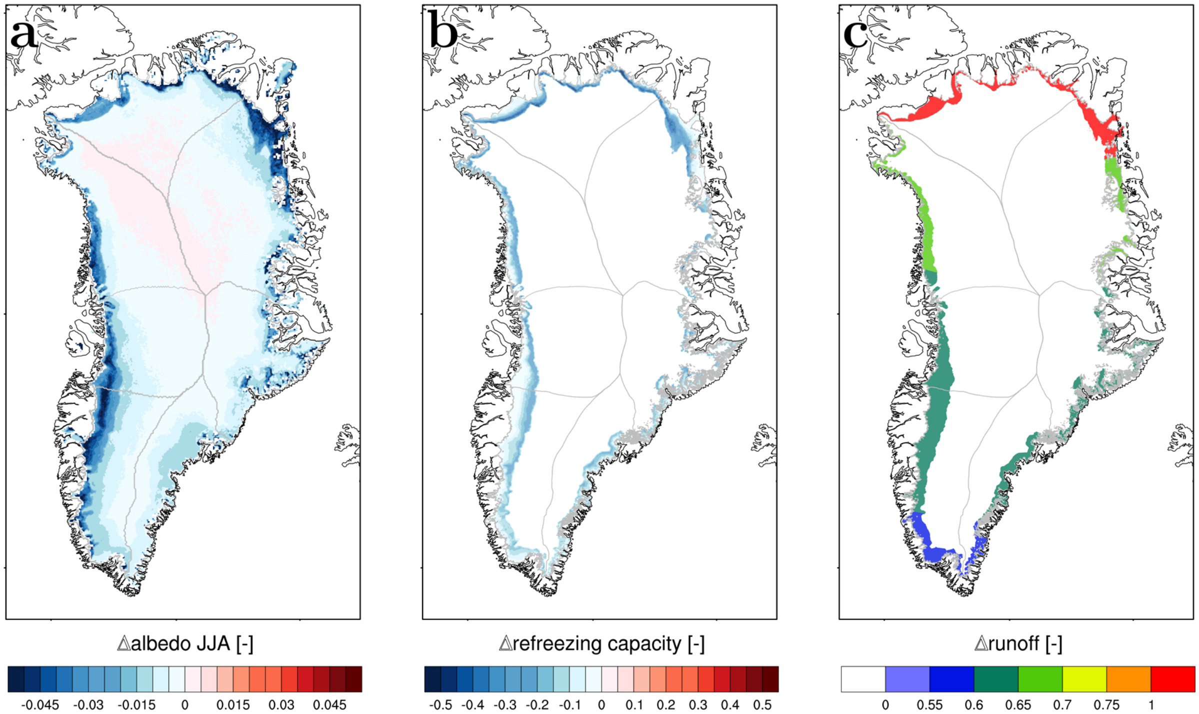

Fig. S9. Post-1990 changes in surface conditions. Post-1990 change in (a) surface albedo (JJA; 1991-2017 minus 1958-1990), (b) refreezing capacity (annual; 1991-2017 minus 1958-1990). (c) Relative change in runoff after 1991 binned within 5° of latitude bands, illustrating the pronounced latitudinal contrast in runoff increase.
